# Voluntary Wheel Running Does Not Enhance Radiotherapy Efficiency in a Preclinical Model of Prostate Cancer: The Importance of Physical Activity Modalities?

**DOI:** 10.3390/cancers13215402

**Published:** 2021-10-28

**Authors:** Suzanne Dufresne, Cindy Richard, Arthur Dieumegard, Luz Orfila, Gregory Delpon, Sophie Chiavassa, Brice Martin, Laurent Rouvière, Jean-Michel Escoffre, Edward Oujagir, Baudouin Denis de Senneville, Ayache Bouakaz, Nathalie Rioux-Leclercq, Vincent Potiron, Amélie Rébillard

**Affiliations:** 1 Movement, Sport and Health Sciences Laboratory (M2S)-EA7470, University of Rennes, F-35000 Rennes, France; suzanne.dufresne@ens-rennes.fr (S.D.); richard.cindy117@gmail.com (C.R.); arthur.dieumegard@etudiant.univ-rennes2.fr (A.D.); orfila-luz.lefeuvre@univ-rennes2.fr (L.O.); brice.mar34@gmail.com (B.M.); 2 Centre René Gauducheau, Institut de Cancérologie de l’Ouest, F-44805 Saint Herblain, France; Gregory.Delpon@ico.unicancer.fr (G.D.); Sophie.Chiavassa@ico.unicancer.fr (S.C.); 3 IRMAR-UMR CNRS 6625, University of Rennes, F-35000 Rennes, France; laurent.rouviere@univ-rennes2.fr (L.R.); vincent.potiron@univ-nantes.fr (V.P.); 4 UMR 1253, iBrain, INSERM, Université de Tours, F-37032 Tours, France; jean-michel.escoffre@univ-tours.fr (J.-M.E.); edward.oujagir@univ-tours.fr (E.O.); ayache.bouakaz@univ-tours.fr (A.B.); 5 Institut de Mathématiques de Bordeaux, UMR 5251, CNRS University of Bordeaux, F-33400 Talence, France; baudouin.denisdesenneville@math.u-bordeaux.fr; 6 Department of Pathological Anatomy and Cytology, Université Rennes 1, F-35000 Rennes, France; Nathalie.RIOUX-LECLERCQ@chu-rennes.fr; 7 LaBCT, CRCINA INSERM U1232, Université de Nantes, Université d’Angers, F-44000 Nantes, France; 8 Institut Universitaire de France (IUF), F-75231 Paris, France

**Keywords:** physical activity, exercise, prostate cancer, radiotherapy, radiation therapy, vascularization, proliferation

## Abstract

**Simple Summary:**

Physical activity is increasingly incorporated in cancer patient health care as a strategy to improve survival outcomes. However, its effects on treatment efficiency remains unclear. The aim of our preclinical study is to evaluate whether access to a running wheel could enhance the response to radiotherapy in mice with prostate cancer. We observed that voluntary wheel running (VWR) did not slow down tumor growth but appeared to modulate some parameters related to tumor perfusion. However, this did not result in enhanced response to radiotherapy. To investigate whether the lack of benefits on tumor growth observed with VWR could be attributed to the choice of physical activity modality, we conducted additional experiments comparing the effects of treadmill running versus VWR in two different preclinical models of prostate cancer. Only treadmill running was able to slow down tumor growth. Hence, the anti-cancer effects of physical activity seem dependent on its modalities.

**Abstract:**

Physical activity is increasingly recognized as a strategy able to improve cancer patient outcome, and its potential to enhance treatment response is promising, despite being unclear. In our study we used a preclinical model of prostate cancer to investigate whether voluntary wheel running (VWR) could improve tumor perfusion and enhance radiotherapy (RT) efficiency. Nude athymic mice were injected with PC-3 cancer cells and either remained inactive or were housed with running wheels. Apparent microbubble transport was enhanced with VWR, which we hypothesized could improve the RT response. When repeating the experiments and adding RT, however, we observed that VWR did not influence RT efficiency. These findings contrasted with previous results and prompted us to evaluate if the lack of effects observed on tumor growth could be attributable to the physical activity modality used. Using PC-3 and PPC-1 xenografts, we randomized mice to either inactive controls, VWR, or treadmill running (TR). In both models, TR (but not VWR) slowed down tumor growth, suggesting that the anti-cancer effects of physical activity are dependent on its modalities. Providing a better understanding of which activity type should be recommended to cancer patients thus appears essential to improve treatment outcomes.

## 1. Introduction

Over the past 20 years, the relationship between physical activity and survival in cancer patients has raised considerable interest [1]. In recent systematic reviews and meta-analyses, it was highlighted that engaging in regular physical activity results in lower all-cause and cancer-specific mortality across several cancer types [2,3]. In line with this, numerous preclinical studies investigated the potential anti-cancer effect of physical activity in a wide variety of cancer types, with the majority reporting reduced tumor initiation or multiplicity and slower tumor growth in active versus sedentary rodents [4]. Importantly, these effects are likely to be dependent on the cancer type or subtype and the physical activity modality used [4]. Nevertheless, several plausible biological mechanisms underpinning the benefits of physical activity on tumor growth have been identified and can be grouped into four main categories: (a) vascularization and blood perfusion, (b) immune function, (c) tumor metabolism and (d) muscle-to-cancer cross-talk [5]. The identification of these mechanisms, as well as the gathering of epidemiological evidence showing improved survival rates in active versus inactive cancer patients, provides a rationale for the potential of physical activity to improve cancer treatment efficiency [6]. In particular, exercise may enhance the delivery of various treatments through increased tumor perfusion and reduced hypoxia. Indeed, two preclinical studies reported that improved tumor vascularization in response to regular physical activity sensitized tumor cells to chemotherapeutic agents in models of breast cancer [7] and pancreatic ductal adenocarcinoma [8]. Furthermore, physical activity has been shown to improve immunotherapy and/or radiotherapy (RT) efficiency through modulation of the immune system in preclinical models of breast and prostate cancer (PCa) [9,10]. However, the potential implication of tumor vascularization in these effects remains unknown. Hence, the aim of the current study is to evaluate whether voluntary wheel running (VWR) was able to improve tumor perfusion and modulate radiotherapy efficiency in a murine model of PCa.

## 2. Materials and Methods

### 2.1. Cell Culture

The PC-3 human prostate cancer cells were either purchased from Sigma-Aldrich (Sigma-Aldrich, St. Louis, MO, USA) or from ATCC (ATCC, Manassas, VA, USA). The PPC-1 human prostate cancer cells were obtained as a gift from Pr. James Norris (Medical University of South Carolina, Charleston, SC, USA). Both prostate cancer cell lines were maintained in a humidified atmosphere at 37 °C; 5% CO_2_. PC-3 cells were cultured in either Coons Modified Ham’s F12 or RPMI 1640 medium, and PPC-1 cells were cultured in RPMI 1640 medium. Medium of both cell lines was supplemented with 10% FBS and 0.1% penicillin/streptomycin (Gibco, Invitrogen, Waltham, MA, USA).

### 2.2. Animals and Tumor Models

Animal experiments were approved by the Animal Experimentation Ethics Committee and were conducted at the animal facility of the M2S laboratory (n°A3504734) in accordance with the ethical standards of the European Community (directive 86/609/EEC). Six seven-week-old male nude athymic mice (Janvier Labs, Le Genest-Saint-Isle, France and Envigo, Gannat, France) were housed in standard laboratory conditions and maintained on a 12:12 h dark–light cycle with a temperature- and humidity-controlled environment. Their body weight was monitored throughout the entire study.

In the first set of experiments, mice received either a subcutaneous injection of 2.5 × 10^6^ human PCa PC-3 cells or PBS. Approximately 5% of the mice injected with PC-3 cells did not develop tumors or had uncharacteristic tumor size/shape (e.g., two separate tumors) and were withdrawn from the study. 24 days following the injection, mice with PC-3 tumors were randomized to either sedentary control or were housed with running wheels. Hence, a total of three experimental groups were determined: (1) Healthy (*n* = 9), (2) Cancer control (CTL, *n* = 8) and (3) Cancer VWR (VWR, *n* = 7). Mice in the VWR group had access to the wheels for a total of 25 days and all the animals included in this set of experiments were euthanized 50 days after cancer cell injection.

In the second set of experiments, mice were also injected subcutaneously with 2.5 × 10^6^ PC-3 cells and randomized 24 days after cancer cell injection to either (1) sedentary controls (CTL; *n* = 9), (2) VWR (*n* = 8), (3) RT (*n* = 9), or (4) RT combined with VWR (RT-VWR; *n* = 9). Mice undergoing VWR had access to a wheel for 25 days, and all animals were euthanized 50 days after cancer cell injection.

In the third set of experiments, mice were injected subcutaneously with either 2.5 × 10^6^ PC-3 cells or 8 × 10^6^ PPC-1 cells. Mice with no tumors or defective tumor growth were withdrawn from the study. PC-3 and PPC-1 mice were randomized 14 days and 7 days following cancer cell injection, respectively. Mice were randomized to either (1) sedentary cancer control (CTL; *n* = 9 for PC-3 and *n* = 9 for PPC-1), (2) VWR (*n* = 7 for PC-3 and *n* = 10 for PPC-1), or (3) treadmill running (TR) (*n* = 9 for PC-3 and *n* = 12 for PPC-1). VWR and TR activities were performed for a total of 50 days in the PC-3 model and 19 days in the PPC-1 model. PC-3 and PPC-1 mice were euthanized 65 days and 27 days following tumor cell injection, respectively.

Tumor volume was assessed regularly throughout the protocol with sliding calipers. The formula used to calculate tumor volume was: length × width × height × 0.5236 (in mm^3^).

At the end of the protocols, mice were anesthetized by intraperitoneal injection of ketamine (50 mg/kg) and xylazine (4 mg/kg), before being euthanized.

Tumors were retrieved and washed in PBS before being separated in two in their center, with half being frozen in liquid nitrogen and the other half being embedded in OCT prior to being immersed in cooled isopentane. Samples were stored at −80 °C.

### 2.3. Physical Activity Models

Mice undergoing VWR activity were housed with running wheels (Intellibio, France) which could be accessed at all times every day. The daily distance covered was calculated and recorded daily. To be able to precisely determine the VWR activity of each mouse, all the animals included in the study were housed individually (including non-VWR mice to avoid bias).

Mice following a TR program ran on a treadmill (Ugo Basile, Gemonio, Italy) five days per week. The intensity and duration of the running sessions reached 18 m/min for 60 min with a 10% incline on day 22. For the final nine sessions, duration was reduced to 45 min/day as the tumor grew larger.

### 2.4. Radiotherapy Treatment

RT treatment was initiated 28 days following cancer cell injection. The system used (XRAD 225Cx, PXI, CRCNA, Nantes, France) has been previously described [10]. Irradiations were delivered with two opposed beams (225 kVp, 13 mA, 0.3 mm Cu filter) and a dose rate of 3.06 Gy/min. The treatment was targeted to the tumor by 3D imaging (Cone Beam CT). The mice undergoing RT received four sessions of 5 Grays (Gy) spread across two weeks (20 Gy total) and were anesthetized upon each RT sessions with 3% halothane in 100% oxygen.

### 2.5. Assessment of Microbubble Transport in the Tumor

In the first set of experiments, four representative mice in the CTL and VWR groups were selected for the assessment of microbubble transport in the tumor. As previously described [11], ultrasound B-mode imaging (Vevo 2100 System, Visualsonics Inc., Toronto, ON, Canada) at 21 MHz (MS-250 probe) was used to image subcutaneous PC-3. Contrast-enhanced ultrasound (CEUS) imaging was performed in order to investigate the tumor perfusion 47 days following PC-3 tumor cell injection. Briefly, a bolus injection of 70 μL of gas microbubbles (Vevo MicroMarker^®^ contrast agents, Visualsonics Inc., Toronto, ON, Canada) was administered intravenously under anesthesia. Immediately after this injection, video clips of nonlinear contrast images were recorded during 36 s at 10 frames/s. The apparent microbubble transport parameters visible on CEUS were quantitatively analyzed as previously reported [12]. The used tool embeds an “optical flow” algorithm [13,14] designed to mimic the human visual perception of object transport in image series. We quantify divergence (sources and sinks) and amplitudes in obtained dense transport fields, which provided very simple indicators of displacement vector orientations and magnitudes [15].

### 2.6. Immunohistochemistry Analyses

Half of each tumor was embedded in OCT prior to being immersed in cooled isopentane and stored at −80 °C. Tissue sections of 8 μm thickness were obtained using the LEICA CM3050S cryostat and mounted on glass slides.

To evaluate tumor cell characteristics, tumor sections were stained with hematoxylin and eosin (H&E) which colors the nucleus in dark purple and the cytoplasm in a lighter pink shade; his enables the evaluation of necrotic areas. Necrotic areas were defined on the H&E slides by the absence of tumor cell nuclei and were expressed as necrotic area relative to total tumor area.

Immunohistological staining for Ki67 was performed with the Ventana detection kit (Ventana Medical Systems, Tucson, AZ, USA) on the Discovery XT Automated IHC stainer as previously described [10]. Slides were incubated with the primary antibody for Ki67 (NB600-1252, Novus Biologicals, Centennial, CO, USA; dilution at 1/400) before using a goat anti-rabbit antibody. A DAPI staining was also performed and cover-slipped. Ki67 was analyzed with ImageJ software and reported as the percentage of Ki67 positive cells relative to the total number of cells.

Immunohistochemistry for tumor vascularization was performed using previously published staining procedures [16]. The following antibodies were used: rat anti-mouse CD31 (BD Biosciences, Le Pont-de-Claix, France), rabbit anti-mouse desmin (Ozyme, Saint-Cyr L’école, France), Cy3-conjugated mouse anti-alpha smooth muscle actin (Sigma-Aldrich), Alexa^647^-conjugated goat anti-rabbit, and Alexa^488^-conjugated goat anti-rat (Life Technologies, Carlsbad, CA, USA). Slides were mounted in Prolong Gold with DAPI (Life Technologies) for nuclei counterstaining and observed using a Vectra (Akoya Biosciences, Marlborough, MA, USA) multispectral fluorescence microscope at 40×. Analyses were performed on original 16-bit tiff images at 40× resolution using ImageJ 1.46r software (National Institutes of Health, Bethesda, MD, USA) as previously described [16]. Vessels were counted as groups formed by CD31-positive pixels distant of ≤1 µm. Alpha-SMA and desmin were measured within the perivascular area, as defined as the 2 µm region surrounding CD31-positive objects.

### 2.7. Western Blot Analyses

Tissue samples were lysed in buffer (50 mM Tris-HCl, 10 mM EDTA, 1% SDS) and protein concentration was determined using a Lowry protein assay. Proteins (50 μg) were separated by SDS-PAGE and transferred onto nitrocellulose membranes (Bio-Rad, Hercules, CA, USA). After being blocked with 5% BSA or nonfat dry milk in TBS-Tween (0.05%), membranes were incubated overnight at 4 °C with the appropriate primary antibodies (Table 1). After being washed three times with TBS-Tween (0.05%), membranes were incubated with secondary antibodies for 1 h at room temperature. Immunoreactive bands were visualized with Odyssey Infrared Imaging System (LI-COR Biosciences, Lincoln, NE, USA). The intensity of each band was quantified with ImageJ (U.S. National Institutes of Health, Bethesda, MD, USA) and normalized to its respective HSC70 band for protein loading. The data was then normalized to the average of the CTL group.

### 2.8. Statistical Analyses

To evaluate the effect of the different strategies on tumor growth, a linear mixed model [17] was used. Indeed, due to repeated measures, we cannot consider that all tumor volumes are independent since many measures are made on the same individuals. Classical linear or ANOVA models are thus not suitable and linear mixed models are usually developed for such data. These models are expanded into two parts: a fixed part which represents global effects of the covariates and a random part which allows avoidance of the problem of non-independence. For our data, we consider:

(1) $$\left[Y_{\left\{itk\right\}}=\alpha_{\left\{0k\right\}}+\alpha_{\left\{1k\right\}t}+\alpha_{\left\{2k\right\}t}^{2}+\beta_{\left\{0i\right\}}+\beta_{\left\{1i\right\}t}+\beta_{\left\{2i\right\}t}^{2}+\varepsilon_{\left\{itk\right\}\;}\right]$$

where *Y_{itk}_* stands for the tumor volume at date *t* for individual *i* with condition *k* (e.g., CTL or VWR). Fixed effects are represented by parameters α, while random effects are collected in *β* parameters. The random parameters (*βi* = (*β*{0*i*}, *β*{1*i*}, *β*{2*i*}), *i* = 1, …, *nI*) are independent and follow a normal distribution with 0 mean and diagonal covariance matrix. Error terms are assumed to be independent with normal distribution. All other statistical analyses were performed in Prism6 (GraphPad software, San Diego, CA, USA). After testing for normality, the results were analyzed using either the t-test or Mann–Whitney test when two groups were compared. When three groups were compared, a one-way ANOVA or Kruskal–Wallis test was used, depending on whether the data followed a normal distribution or not, followed by Tukey or Dunn’s post hoc tests, respectively. In the set of experiments combining VWR with RT (with a total of four experimental groups), the data was analyzed using a two-way ANOVA with the two main variables being (a) VWR and (b) RT. If the two-way ANOVA showed a significant interaction, the main effects were analyzed, and Tukey post hoc tests were performed. Finally, tumor growth was assessed using a mixed model. Comparisons were considered significant for *p* values below 0.05. Data are represented as means ± SEM.

## 3. Results

### 3.1. VWR Alters Microbubbles Apparent Transport in the Tumor Tissue

**Figure 1:**
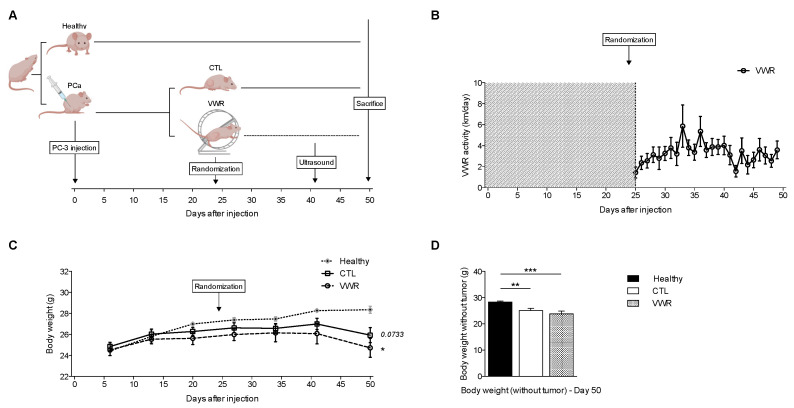
PC-3 xenografts exhibit lower body weight than non-tumor-bearing nude mice and VWR does not counteract this effect. (**A**) Experimental design; (**B**) VWR activity (in km/day) over time; (**C**) Body weight (in g) evolution; (**D**) Body weight after tumor was retrieved (in g) the day mice were euthanized (day 50). Data are represented as means ± SEM. * *p* < 0.05; ** *p* < 0.01; *** *p* < 0.001.

The experimental protocol performed to assess whether VWR could impact microbubble transport is depicted in Figure 1A. In these experiments, PC-3 mice ran on average 3.13 ± 0.68 km/day (Figure 1B) and this activity did not result in body weight differences compared to tumor-bearing CTL mice. However, both inactive and active tumor-bearing mice exhibited lower body weight compared to healthy mice (Figure 1C,D).

We firstly evaluated whether VWR initiated in established tumors was able to modulate tumor perfusion in a PC-3 xenograft model.

**Figure 2:**
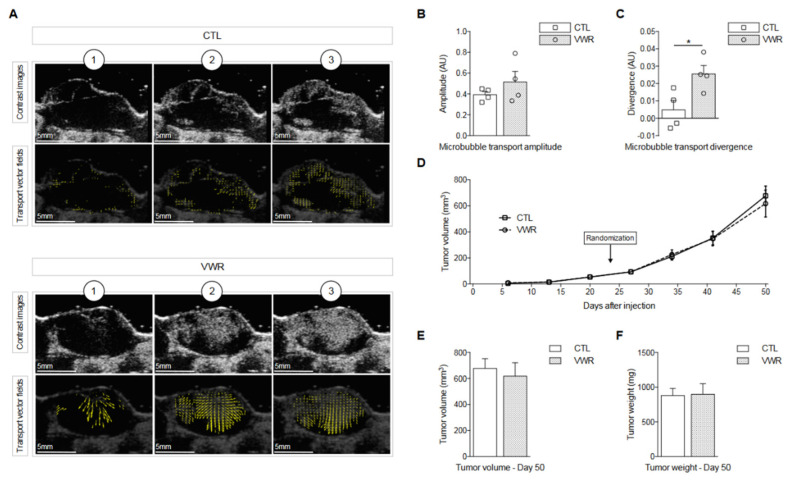
VWR modulates microbubble transport in the tumor but does not impact tumor growth. (**A**) Representative images of microbubble transport estimates from the DCEUS in CTL and VWR mice acquired at (1) 0 s, (2) 1 s and (3) 2 s after bolus arrival. For each experimental group, the first row shows contrast images and the second row displays estimated apparent transport fields; (**B**) Quantification of microbubble transport amplitude; (**C**) Quantification of microbubble transport divergence; (**D**) Tumor volume (in mm^3^) evolution; (**E**) Tumor volume (in mm^3^) on the day mice were euthanized (day 50); (**F**) Tumor weight (in mg) on day 50. Data are represented as means ± SEM. * *p* < 0.05.

To gather information related to tumor perfusion, dynamic contrast agent enhanced ultrasound was performed 41 days after tumor cell injection with gas-filled microbubble contrast agents used as intravascular flow tracers. Both apparent microbubble transport amplitude and divergence evident on CEUS were analyzed. The amplitude corresponds to microbubble apparent velocity, while transport field divergence represents the directions of tumor blood flow (i.e., sources/centripetal trajectories in estimated velocity vector fields). The results obtained showed that microbubble transport amplitude was unaffected by VWR (Figure 2A,B), while transport field divergence was significantly increased with VWR (*p* < 0.05; Figure 2A,C), suggesting modifications of tumor perfusion with VWR.

### 3.2. VWR Does Not Impact PC-3 Tumor Growth

Despite the differences observed on the tumor ultrasound, VWR did not slow down tumor growth (Figure 2D). On the last day of the experiment (day 50), average tumor volume was 677 ± 75 mm^3^ in CTL mice and their tumors weighed 880 ± 103 mg. VWR animals had tumors measuring 620 ± 90 mm^3^ and weighing 871 ± 136 mg on average, with no notable difference compared to the CTL group (Figure 2E,F).

### 3.3. VWR Does Not Impact RT Efficiency in PC-3 Xenografts

**Figure 3:**
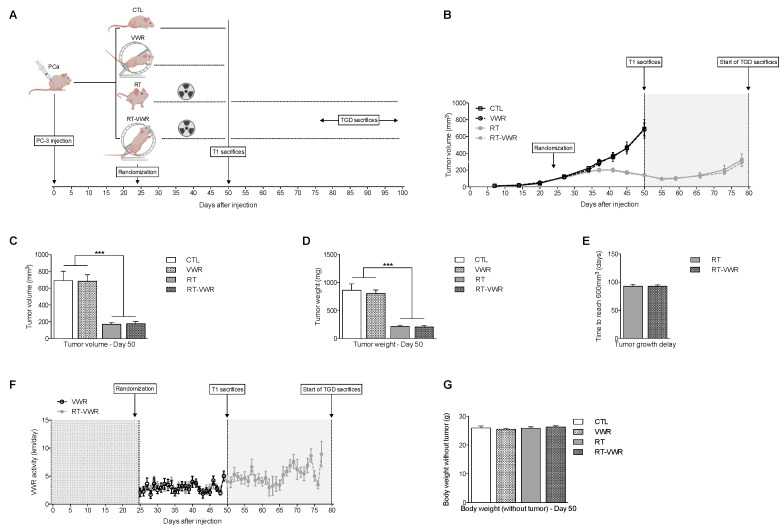
VWR combined with RT does not affect treatment efficiency. (**A**) Experimental design; (**B**) Tumor volume (in mm^3^) evolution; (**C**) Tumor volume (in mm^3^) of mice euthanized at T1 (day 50); (**D**) Tumor weight (in mg) of mice euthanized on day 50; (**E**) Number of days for the tumor to reach 600 mm^3^ in RT and RT-VWR following RT treatment (tumor growth delay); (**F**) VWR activity over time (in km/day); (**G**) Body weight after tumor was retrieved (in g) at T1 (day 50). Data are represented as means ± SEM. *** *p* < 0.001.

Modulation of tumor perfusion can improve tumor oxygenation, which is a critical factor for radiotherapy efficiency [18]. Since it appears that tumor perfusion was modulated by VWR, we conducted a second set of experiments to assess whether VWR could enhance RT efficiency (the study design can be found on Figure 3A). We confirmed that VWR did not influence tumor growth (Figure 3B), with tumors averaging 684 ± 77 mm^3^ and 802 ± 63 mg in VWR mice compared to 691 ± 111 mm^3^ and 860 ± 116 mg in the control group (Figure 3C,D). RT, as expected, decreased tumor growth (Figure 3B) with tumors reaching 172 ± 19 mm^3^ and 214 ± 25 mg on day 50. The combination of RT with VWR did not influence RT efficiency (VWR main effect *p* > 0.05; RT main effect *p* < 0.001) with the tumors of RT-VWR mice measuring 177 ± 27 mm^3^ and 205 ± 29 mg (Figure 3C,D). To evaluate whether this lack of effect could be attributable to a relatively short VWR period (25 days), we analyzed tumor relapse following RT, keeping RT and RT-VWR mice for an additional 23–51 days (48–76 days of VWR total) until tumor volume reached ∼600 mm^3^ after RT cessation (Figure 3A). The time for the tumor to reach 600 mm^3^ (tumor growth delay) was, however, similar between RT and RT-VWR mice, both averaging ∼93 days after injection (Figure 3E), confirming that in our model, VWR does not impact RT efficiency. Overall, VWR post-tumor establishment had no effect on PC-3 tumor growth, whether performed alone or when combined with RT.

### 3.4. VWR Is Not Affected by RT and Does Not Result in Weight Loss

To evaluate whether the lack of effect of VWR on RT efficiency could be attributable to lower VWR activity in RT mice, the distance run by each mouse was recorded daily. On average, VWR mice ran 2.92 ± 0.55 km/day (similar to what was found in the first set of experiments) and RT-VWR mice 3.32 ± 0.40 km/day, with no significant differences observed between these two groups (Figure 3F). Hence, RT did not impact VWR activity. Of note, none of the interventions resulted in significant weight loss compared to cancer control mice (Figure 3G).

### 3.5. RT but Not VWR Is Effective at Remodeling Tumor Vasculature

**Figure 4:**
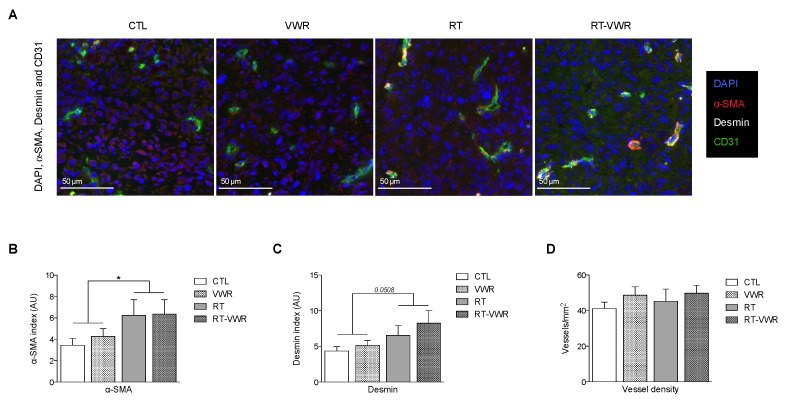
VWR does not provide additional benefits to RT-induced modulation of tumor vasculature. (**A**) Representative images of DAPI (blue), α-SMA (red), desmin (white) and CD31 (green) staining; (**B**) Quantification of α-SMA staining; (**C**) Quantification of desmin staining; (**D**) Quantification of vessel density (vessel/mm^2^). Data are represented as means ± SEM. * *p* < 0.05.

Tumor vascularization is dependent on the functionality but also on the morphology of tumor vessels. In the first set of experiments, we observed enhanced microbubble transport divergence with VWR, but the morphology of tumor vasculature was not evaluated. To assess this parameter, immunostaining of pericyte markers α-smooth-muscle-actin (α-SMA) and desmin were performed in the tumor tissue, and vessel density was measured. Neither of these markers were affected by VWR, while RT resulted in a significant increase in α-SMA (RT main effect *p* < 0.05; Figure 4B) and a borderline increase in desmin (RT main effect *p* = 0.0508; Figure 4A,C). Vessel density was similar across all groups (Figure 4A,D). Hence, only RT appeared to remodel tumor vasculature by increasing tumor pericyte coverage, while VWR did not provide additional effects. The number of vessels remained unchanged in response to either VWR or RT. The improved tumor vascularization measured in the first set of experiments are therefore likely a result of improvements in tumor vessel functionality rather than changes in their morphology.

### 3.6. VWR Does Not Influence the RT-Induced Inhibition of Cell Proliferation and Survival

**Figure 5:**
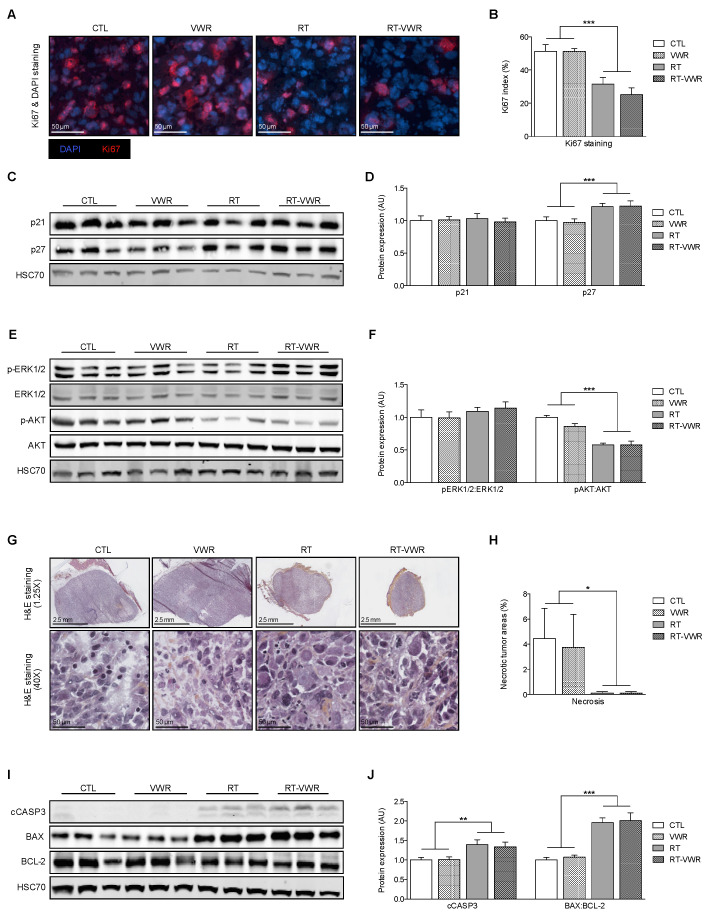
VWR does not modulate the effects of RT on tumor cell death, proliferation and survival. (**A**) Representative images of Ki67 (red) and DAPI (blue) staining; (**B**) Percentage of Ki67 positive cells; (**C**) Representative Western blot images of p21 and p27 proteins; (**D**) Quantification of p21 and p27 protein expression; (**E**) Representative Western blot images of p-ERK1/2, ERK1/2, p-AKT, and AKT proteins; (**F**) Quantification of p-ERK1/2, ERK1/2, p-AKT, and AKT protein expression; (**G**) Representative images of H&E staining; (**H**) Percentage of necrotic areas; (**I**) Representative Western blot images of cCASP3, BAX and BCL-2 proteins; (**J**) Quantification of cCASP3 and BAX:BCL-2 protein expression. Data are represented as means ± SEM. * *p* < 0.05; ** *p* < 0.01; *** *p* < 0.001.

Even though prostate tumors did not differ in volume or weight, we evaluated whether VWR alone or combined with RT could alter tumor physiology and signaling pathways. Ki67/DAPI co-staining was performed on tumor sections of mice sacrificed at T1. The images revealed that VWR did not affect Ki67 index, while it was reduced in both RT and RT-VWR mice by 39% and 51%, respectively. There was no significant interaction effect (*p* = 0.4317), revealing that the combination of RT and VWR did not significantly decrease Ki67 staining compared to RT alone (VWR main effect *p* > 0.05; RT main effect *p* < 0.001; Figure 5A,B).

At the molecular level, the expression of key cell cycle regulators p21 and p27 was assessed in the tumor tissue. None of the interventions affected p21 expression (VWR main effect *p* > 0.05, RT main effect *p* > 0.05; Figure 5C,D). p27 was not affected by VWR, but increased by 22% in both RT and RT-VWR (VWR main effect *p* > 0.05; RT main effect *p* < 0.001; Figure 5C,D). Therefore, only RT was able to modulate cell cycle regulators.

The activation of ERK1/2 and AKT were also evaluated, as AKT/mTOR and ERK MAPK signaling pathways are frequently hyperactivated in castration resistant PCa, where they promote proliferative and survival pathways [19,20]. In our study, the pERK1/2:ERK1/2 protein ratio was similar across all experimental groups. The pAKT:AKT protein ratio remained unchanged with VWR but was significantly reduced by 42% in both RT and RT-VWR mice. VWR combined with RT did not provide any additional effect compared to RT alone (VWR main effect *p* > 0.05; RT main effect *p* < 0.001; Figure 5E,F). Hence, neither VWR nor RT was able to modify the activation of ERK1/2, but RT significantly decreased AKT activation.

In summary, only RT reduced the rate of cell division and lowered AKT signaling, with no added benefits from VWR. All samples run for Western blots can be found in Figure S1.

### 3.7. VWR Does Impact Tumor Cell Death, Even When Combined with RT

To evaluate the impact of the different interventions on cell death, both necrosis and apoptosis were assessed. An H&E staining was performed on tumor sections and showed that the percentage of tumor necrotic areas was similar between CTL and VWR mice (representing 4.44% and 3.75% of the total tumor area in CTL and VWR mice, respectively), while they were completely non-existent in both RT and RT-VWR groups (VWR main effect *p* > 0.05; RT main effect *p* < 0.05; Figure 5G,H). Apoptosis signaling was analyzed by Western blot with antibodies directed against cleaved caspase-3 (cCASP3), BAX and BCL-2 proteins. Apoptotic effector cCASP3 protein expression remained unchanged with VWR, but was significantly increased with RT by 40%. VWR combined with RT resulted in a similar increase (34%) compared to RT alone (VWR main effect *p* > 0.05; RT main effect *p* < 0.01; Figure 5I,J). Similarly, BAX:BCL-2 protein ratio was not affected by VWR but increased in both RT groups (by 96% and 100% in RT and RT-VWR mice, respectively), with no added benefits from VWR (VWR main effect *p* > 0.05; RT main effect *p* < 0.001; Figure 5I,J).

Therefore, VWR did not impact cancer cell death, while RT lowered necrosis and enhanced apoptosis, regardless of VWR.

In summary, VWR did not impact tumor growth nor RT efficiency, and did not appear to modulate major tumor signaling pathways. This contrasts with previous findings published by our team [10]. Importantly, two major factors differ between the two studies: the physical activity modality (treadmill running (TR) versus VWR) and the cancer cell line used (PPC-1 versus PC-3). To evaluate whether the absence of anti-cancer effects observed with VWR in PC-3 xenografts could be attributed to the choice of physical activity modality, we investigate the effect of VWR versus TR in both PC-3 and PPC-1 xenografts.

### 3.8. TR, But Not VWR, Slows Down Tumor Growth in Both PC-3 and PPC-1 Xenografts

**Figure 6:**
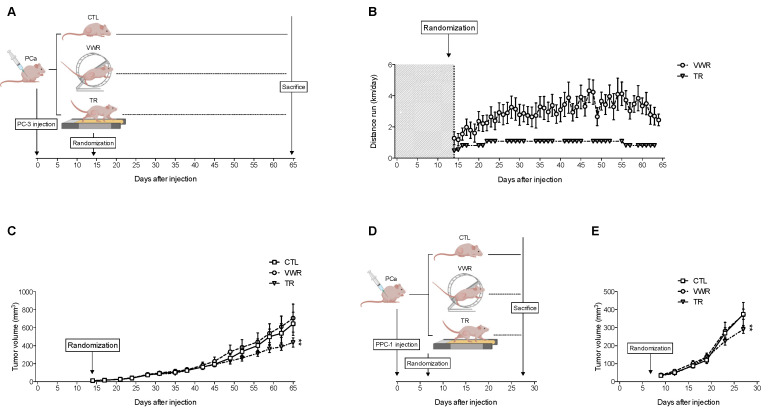
TR slows down tumor growth in both PC-3 and PPC-1 xenografts, while VWR has no effect. (**A**) Experimental design with PC-3 xenografts; (**B**) Distance ran (km/day) throughout the protocol by VWR and TR mice; (**C**) Tumor volume (in mm^3^) evolution in PC-3 xenografts; (**D**) Experimental design with PPC-1 xenografts; (**E**) Tumor volume (in mm^3^) evolution in PPC-1 xenografts. Data are represented as means ± SEM. ** *p* < 0.01.

To investigate this hypothesis, we conducted another set of experiments where the effect of TR and VWR on tumor growth was assessed within the same tumor model (PC-3 xenografts) described in Figure 6A.

Here, VWR mice ran an average of 3.93 ± 0.57 km/day, and hence ran approximately 27.5 km/week. TR mice on the other hand ran five days/week for up to ∼1.08 km/day (18 m/min with 10% incline for one hour) corresponding to 5.4 km/week (Figure 6B).

Tumor volume was regularly assessed throughout the protocol (similar to previous experiments) to evaluate PCa growth. Interestingly, when using linear mixed models, we observed that TR had a significant effect on tumor growth (Figure 6C), while VWR did not (similar to what was observed in previous experiments). When repeating the experiments in a different preclinical prostate cancer model using PPC-1 xenografts (Figure 6D), we observed similar effects (Figure 6E).

Hence, the anti-cancer effects of physical activity in both PC-3 and PPC-1 xenografts appear to be dependent of the modalities used, with TR being effective at slowing down tumor growth but not VWR. This effect is not attributable to greater distance ran as VWR mice covered approximately five times longer weekly distances than TR mice.

## 4. Discussion

Our study is the first to investigate the impact of VWR (initiated after tumor establishment) on the RT response. The main findings obtained are (1) that VWR activity was only able to modulate some parameters related to tumor perfusion (e.g., VWR enhanced the divergence of the apparent microbubble transport field but did not impact its amplitude), but neither tumor growth nor RT efficiency was influenced by VWR; and (2) the lack of effects observed on tumor growth could be attributable, at least in part, to the choice of physical activity modality, as TR—unlike VWR—was able to slow down prostate tumor growth.

### 4.1. VWR Does Not Slow Down Tumor Growth but Modulates Microbubble Transport in the Prostate Tumor Tissue

In our study, VWR did not impact tumor growth. These results are in contrast with studies previously published in the literature. Indeed, the majority of preclinical studies investigating the effect of physical activity on tumor growth reported slower tumor growth in active rodents [4]. Nevertheless, there are large disparities between studies, with some reporting no effect or even increased tumor growth in response to physical activity. In PCa more specifically, some studies reported slower tumor growth with physical activity [10,21,22,23,24,25,26,27], while others found no effect [27,28,29,30,31,32,33,34]. McCullough et al. even reported that while TR had no effect on tumor weight in immunocompetent Copenhagen rats, it significantly increased tumor weight in nude rats [28]. These discrepancies may be attributed to differences in the physical activity models used (i.e., modality, dose, timing) or in the cancer models employed (i.e., host, tumor induction, tumor type and subtype, site of tumor development) [4]. Interestingly, Zheng et al. used a similar cancer model to ours (PC-3 xenograft) and showed that VWR activity was able to reduce PCa growth [21]. Importantly, however, VWR was initiated one week prior to tumor cell injection, while in our study, VWR started 14 days post-injection at the earliest. The timing of VWR may be important as engaging in regular physical activity before tumor cell inoculation could notably prepare the tissue microenvironment, and more broadly the host, against future tumor formation. However, in our study we chose to focus on physical activity initiated after tumor establishment as it may be partially representative of previously sedentary PCa patients engaging in regular physical activity after diagnosis as part of their health care.

Nevertheless, despite no effect on tumor growth being observed, VWR was able to enhance the divergence of the apparent microbubble transport, suggesting enhanced blood flow distribution. These results are not surprising as there is accumulating evidence showing that physical activity is able to improve tumor vascularization, as reviewed and discussed elsewhere [35,36]. In PCa more specifically, both acute and chronic physical activity have been shown to modulate tumor perfusion/vascularization. Indeed, an acute bout of treadmill running resulted in increased tumor blood flow (by approximately 180–200%) and resulted in a higher number of patent vessels compared to what was measured at rest in rats bearing orthotopic prostate tumors [37,38]. Chronic physical activity following PCa transplant has also been shown to improve prostate tumor vascularization in two preclinical studies [28,29], which is in line with our findings.

Tumor growth relies partly on angiogenesis, by which new blood vessels are created to grant oxygen and nutrients to the tumor. However, aberrant angiogenic signaling leads to a chaotic and immature vessel structure. As a result, a discrepancy between the oxygen supply and demand occurs, thereby resulting in hypoxia [39]. In PCa, hypoxia has been shown to correlate with increased tumor aggressiveness, higher metastatic potential, and poor prognosis [40]. Hence, improving tumor perfusion with VWR may result in tumor reoxygenation and improve PCa outcomes. However, in our study, there was no difference in tumor growth between sedentary and VWR mice. Nevertheless, improved tumor perfusion associated with VWR may limit the establishment of hypoxia, known to be associated with radioresistance. Indeed, low oxygen availability reduces the amount of reactive oxygen species induced by radiotherapy, and a hypoxic microenvironment leads to the selection of radioresistant cancer cells [18]. Hence, higher tumor blood flow in response to physical activity may improve tumor oxygenation and enhance treatment response [6,36], similar to what can be observed with hyperthermia [41]. Thus, in our model, we hypothesized that despite no effect of VWR alone on tumor growth, the combination of VWR with RT could improve RT efficiency through improved tumor perfusion.

### 4.2. VWR Does Not Affect Tumor Growth and the RT Response

Contrary to our hypothesis, VWR did not improve RT efficiency as no difference in tumor growth rate, tumor volume, tumor weight nor tumor growth delay was found. This was not related to an impairment of VWR activity by RT, as VWR and RT-VWR mice ran comparable distances throughout the experiment. At the molecular level, VWR did not impact tumor cell proliferation nor cancer cell death, even when combined with RT. Hence, even though VWR appeared safe, it did not alter major tumor characteristics. Importantly, even though microbubble transport divergence was enhanced by VWR, no difference in microbubble transport amplitude, in tumor pericyte coverage, nor in vessel density was found. RT on the other hand led to increased pericyte coverage, suggesting a remodeling of tumor vasculature with RT, as previously reported [16], and VWR did not provide additional effects. Hence, physical activity in our model may have only led to a moderate effect on tumor vascularization. This might notably be attributed to the use of ectopic tumors rather than orthotopic, as the tumor vascularization in response to acute exercise has been shown to be highly dependent on the inoculation site. Garcia et al. indeed reported that while tumor blood flow was increased by ∼180% in rats bearing orthotopic prostate tumors, it was reduced by ∼25% in rats with ectopic tumors [38]. Differences in tumor blood flow between ectopic and orthotopic tumors in response to chronic physical activity were however not investigated. Hence, the impact of improved tumor vascularization with physical activity on RT efficiency remains to be evaluated in an orthotopic PCa model.

Two previous preclinical studies have reported improved RT response with physical activity. Wennerberg et al. found enhanced treatment response with TR in mice treated with RT combined with immunotherapy in a murine model of 4T1 mammary carcinoma [9]. Furthermore, our team recently showed improved RT efficiency with TR in a PCa xenograft model [10], a model relatively similar to the present study. Hence, we conducted additional experiments to better understand whether the physical activity model represents a major determinant for the anti-cancer effects of physical activity.

### 4.3. TR Slows Down PC-3 and PPC-1 Tumor Growth

To assess the importance of physical activity modalities on prostate tumor growth, we evaluated whether TR rather than VWR could alter tumor growth in vivo. TR (but not VWR) significantly slowed down tumor growth rate, suggesting that the anti-cancer effects of physical activity is dependent of the modalities used. Similar results were found when replicating the same experiments in PPC-1 xenografts. These findings could appear to be in contrast with a recent study showing no effect of TR on the prostate tumor weight of rats bearing orthotopic PC-3 tumors [33]. However, in our work, tumors had a similar volume and weight to the ones measured in CTL and VWR mice on the final day of the protocol. This can be explained by the fact that the linear mixed models used to determine tumor growth in our study are more thorough analyses as they take into account the notion of time, while tumor volume and weight measured when mice are euthanized do not take into account the measurements of the previous days.

To understand why two activities had distinct effects on tumor growth in our study, we kept track of the daily distance ran by mice. Interestingly, mice undergoing VWR covered longer distances than mice submitted to TR, which suggests that longer distances are not associated with anti-cancer effects in our model. Another plausible explanation is difference in intensity. Several observational studies indeed support this hypothesis and suggest that exercise intensity rather than duration may improve survival of prostate cancer patients. In particular, Kenfield et al. found an inverse relationship between risk of prostate cancer-specific death and vigorous physical activity [42], while Richman et al. showed that walking pace, but not walking duration, was associated with a decrease in disease progression [43]. It was also shown that men diagnosed with prostate cancer engaging in walking/cycling or exercise had significantly reduced cancer-specific mortality rates, but this effect was not observed for housework or total recreational activity [44]. Another study found that low volumes of recreational activity were associated with decreased prostate cancer mortality, which was not the case for occupational and household activities (even at high volumes), indirectly suggesting that higher intensities are necessary to induce survival benefits [45]. Taken together, these results suggest that intensity could represent a key determinant in the anti-cancer effects of physical activity in prostate cancer, while total duration may not be of great importance. Therefore, targeting a specific heart rate could represent an interesting strategy to maximize the benefits associated with physical activity in PCa patients. However, extrapolating preclinical data to the clinical setting is difficult and whether this mediates the difference of effects between TR and VWR observed in our study is speculative. This deserves additional investigation and developing preclinical models aiming to evaluate the effect of different exercise modalities in combination with conventional treatments on tumor growth is an important first step to better determine the physical activity modalities to recommend for cancer patients.

Gathering such information will enable a move towards the idea of “personalized medicine” for exercise in the oncology setting.

### 4.4. Limitations

It is necessary to discuss that there are some limitations to the present study. Notably, the animals used in our experiments were immunodeficient, in order to evaluate the impact of physical activity on tumors of human origin. Since immunodeficient mice do not benefit from the adaptive immune response, some of the effects of physical activity might be masked.

It is also important to note that “normal” cage activity and energy expenditure were not monitored and therefore potential bias may have been introduced if the CTL mice engaged in important activity within their cage. Furthermore, the effect on VWR and TR on muscular adaptations were not evaluated in this study, which does not allow us to confirm whether these modalities induced physiological effects. It was, however, previously shown that mice undergoing either VWR or TR displayed metabolic adaptations (such increased mitochondria biogenesis in the skeletal muscle), showing that their activity level was higher than the sedentary controls [46].

Finally, tumors were injected subcutaneously, which may not provide the same microenvironment as the one from the tissue of origin (i.e., the prostate).

## 5. Conclusions

Overall, our study showed that VWR initiated after tumor establishment was able to alter some parameters related to tumor perfusion (e.g., tumor flux divergence) in a preclinical model of PCa, but surprisingly influenced neither tumor growth nor RT efficiency. In line with this, no differences were found at the molecular level. We further showed that physical activity might however provide benefits in PCa, but that these effects were dependent on physical activity modality. Indeed, we found that TR was able to slow down tumor growth in mice bearing PC-3 or PPC-1 tumors, while VWR did not.

Thus, this study highlights the necessity to better understand what type of physical activity should be recommended to limit PCa mortality. Interestingly, the American College of Sports Medicine recently provided updated physical activity guidelines for cancer patients where they specified the most effective type of physical activity for improving specific cancer-related symptoms such as fatigue or depression [47]. Future studies evaluating the effects of different physical activity type/modalities on tumor growth could help elaborate similar guidelines to maximize survival benefits.

## Figures and Tables

**Table 1:** List of the antibodies used for Western blot.

Protein	MW ^1^ (kDa)	Reference	Dilution	Source
cCASP3	17, 19	Cell signaling 9661	1/500	Rabbit
BAX	20	Cell signaling 2772	1/1000	Rabbit
P21	21	Cell signaling 2947	1/1000	Rabbit
P27	27	Cell signaling 2552	1/1000	Rabbit
BCL-2	28	Abcam ab7973	1/1000	Rabbit
p-ERK1/2	42, 44	Cell signaling 4376	1/1000	Rabbit
ERK1/2	42, 44	Santa Cruz sc-514302	1/1000	Mouse
p-AKT	60	Cell signaling 9271	1/1000	Rabbit
AKT	60	Cell signaling 9272	1/1000	Rabbit
HSC70	70	Santa Cruz sc-7298	1/5000	Mouse

^1^ Molecular Weight (MW).

## Data Availability

The data presented in this study are available on request from the corresponding author.

## Supplementary Materials

The following are available online at https://www.mdpi.com/article/10.3390/cancers13215402/s1, Figure S1: Full Western blot images for Figure 5A.
