# Supplementary material for: Voluntary Wheel Running Does Not Enhance Radiotherapy Efficiency in a Preclinical Model of Prostate Cancer: The Importance of Physical Activity Modalities?
Source: Cancers (Basel). 2021 Oct 28;13(21):5402. doi: 10.3390/cancers13215402 (PMC8582584; doi:10.3390/cancers13215402)
Supplement: Supplementary file 1 [file cancers-13-05402-s001.zip › cancers-1417427-supplementary.pdf]

Supplementary Materials

Voluntary Wheel Running Does Not Enhance Radiotherapy Efficiency in a Preclinical Model of Prostate Cancer: The Importance of Physical Activity Modalities?

Suzanne Dufresne, Cindy Richard, Arthur Dieumegard, Luz Orfila, Gregory Delpon, Sophie Chiavassa, Brice Martin, Laurent Rouvière, Jean-Michel Escoffre, Edward Oujagir, Baudouin Denis de Senneville, Ayache Bouakaz, Nathalie Rioux-Leclercq, Vincent Potiron and Amélie Rébillard

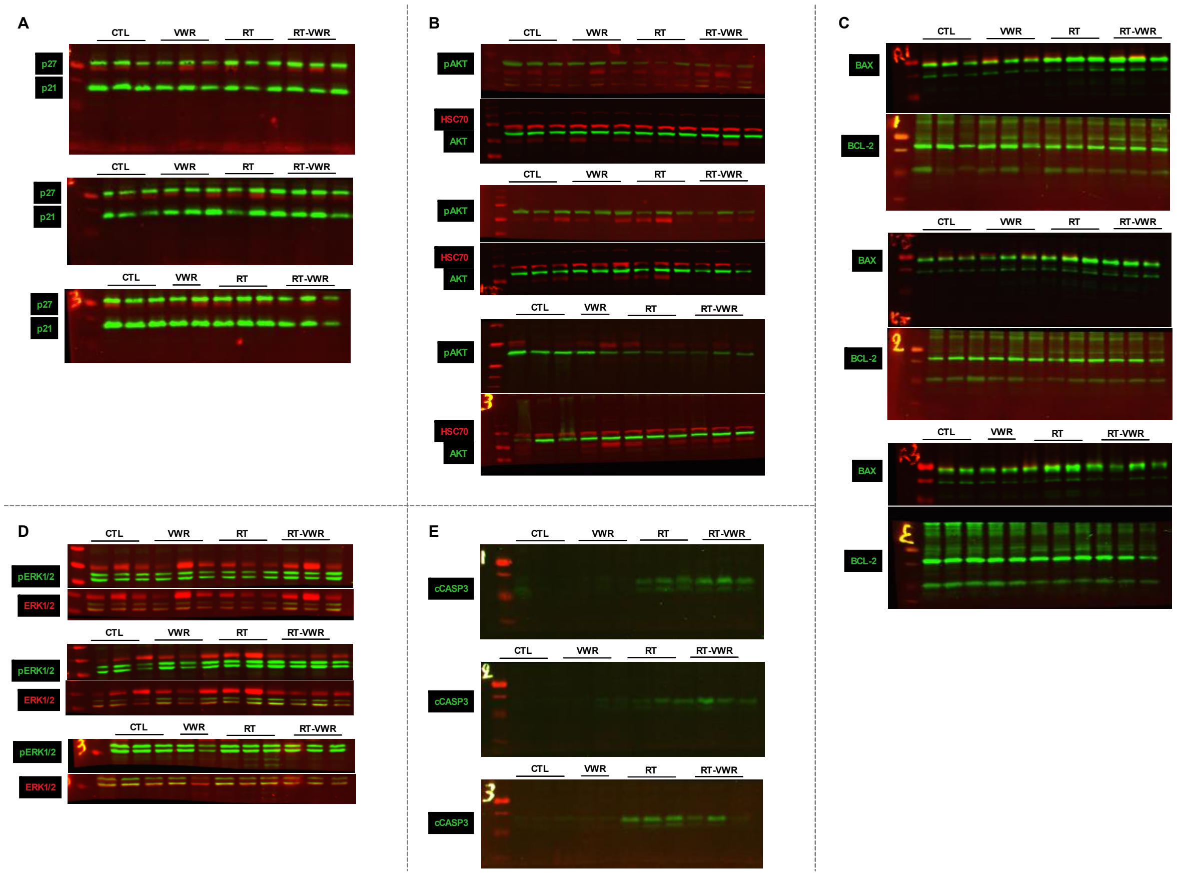

Figure S1. Full Western blot images for Figure 5A. p27 (top green band) and p21 (bottom green band). B. pAKT (green) and HSC70 (red). C. BAX (green) and BCL-2 (green). D. pERK1/2 (green) and ERK1/2 (red). E. cCASP3 (green).

© 2021 by the authors. Licensee MDPI, Basel, Switzerland. This article is an open access article distributed under the terms and conditions of the Creative Commons Attribution (CC BY) license (http://creativecommons.org/licenses/by/4.0/).
